# Protective role of remogliflozin against experimental liver fibrosis by activating AMPK/SIRT1/Nrf2 and suppressing NF-κB pathways

**DOI:** 10.3389/fphar.2025.1586231

**Published:** 2025-06-09

**Authors:** Naif ALSuhaymi, Mahdi H. Alsugoor, Aya A. Shokry, Hany M. Fayed, Bassim M. S. A. Mohamed, Sherif M. Afifi, Tuba Esatbeyoglu, Reda M. S. Korany, Marawan A. Elbaset

**Affiliations:** ^1^ Department of Emergency Medical Services, Faculty of Health Sciences, AlQunfudah, Umm Al-Qura University, Makkah, Saudi Arabia; ^2^ Department of Pharmacology, Faculty of Veterinary Medicine, Cairo University, Cairo, Egypt; ^3^ Department of Pharmacology, Medical Research and Clinical Studies Institute, National Research Centre, Cairo, Egypt; ^4^ Department for Life Quality Studies, Rimini Campus, University of Bologna, Rimini, Italy; ^5^ Department of Molecular Food Chemistry and Food Development, Institute of Food and One Health, Gottfried Wilhelm Leibniz University Hannover, Hannover, Germany; ^6^ Department of Pathology, College of Veterinary Medicine, Cairo University, Cairo, Egypt; ^7^ Stark Neurosciences Research Institute, Indiana University School of Medicine, Indianapolis, IN, United States; ^8^ Department of Neurology, Indiana University School of Medicine, Indianapolis, IN, United States

**Keywords:** liver fibrosis, thioacetamide, Nrf2, SIRT1, oxidative stress, remogliflozin

## Abstract

Liver fibrosis is considered an epidemic health problem since it can lead to several insults that can be fatal. Remogliflozin (Remo), an inhibitor of the sodium–glucose cotransporter 2 (SGLT2) protein, is one of the most recently developed antidiabetic drugs for treating type 2 diabetes mellitus (T2DM). The antidiabetic and antioxidant impacts of Remo have been demonstrated in numerous animal models; however, its antifibrotic activity remains unclear. Therefore, we planned this study to clarify the preventive activity of Remo against thioacetamide (TAA)-induced liver fibrosis in male rats, along with its anticipated pathways. Four groups of rats (n = 6) were used in our investigation: the control group; the TAA group, which received 100 mg/kg b.wt IP twice a week for 6 weeks; and the TAA + Remo groups, which were given two doses of Remo at 25 and 50 mg/kg b.wt orally, respectively, for 4 weeks in addition to TAA injections. The TAA group showed a marked increase in liver enzymes, lipid peroxidation, and proinflammatory cytokines, along with a marked decrease in albumin and cellular antioxidant status. Additionally, the TAA group showed a marked increase in nuclear factor-κB (NF-κB) and a marked decrease in AMP-activated protein kinase (AMPK), sirtuin 1 (SIRT1), and nuclear factor erythroid 2–related factor 2 (Nrf2) levels and expressions. The harmful effects of TAA were significantly mitigated by Remo therapy, which improved the aforementioned parameters. Histopathological findings corroborated the biochemical results. The results of our study suggest that Remo has anti-inflammatory and antioxidant properties that protect against TAA-induced liver fibrosis by inhibiting the NF-κB pathway and activating the AMPK/SIRT1/Nrf2 pathway.

## Graphical Abstract

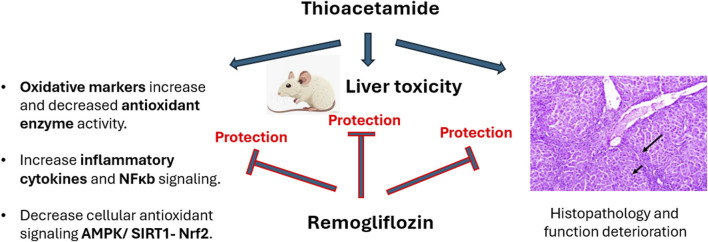

## 1 Introduction

The pathophysiological process of liver fibrosis is complex and constitutes a transitional stage for several chronic liver illnesses. It is typified by abnormal extracellular matrix (ECM) development (Xiu and Ding, 2021). Chronic liver conditions caused by several factors, such as drug overdose, alcoholism, nonalcoholic fatty liver disease (NAFLD), viral hepatitis, and autoimmune conditions, can result in hepatic fibrogenesis. Despite years of intensive research on the biology of hepatic fibrosis, no therapy is currently available (Mallat and Lotersztajn, 2013; Ramadan et al., 2018). Hepatic stellate cells (HSCs) are the main liver cells that generate the extracellular matrix (Mohammed et al., 2023). After being stimulated by fibrogenic factors, HSCs change from vitamin A-storing latent cells to active cells that resemble myofibroblasts, depositing an abundance of ECM, especially type I collagen. These myofibroblasts release several pro-inflammatory and pro-fibrotic cytokines (Abd El-Rahman and Fayed, 2019).

Oxidative stress leads to lipid, protein, and DNA degradation, accelerating hepatic fibrogenesis. It results from an imbalance in the generation and removal of free radicals by cells (Tang et al., 2014). Nuclear factor erythroid 2–related factor 2 (Nrf2) is vital in defending the liver against oxidative injury, during fibrogenesis and inflammation (Abdel-Rahman et al., 2022). Normally, Kelch-like ECH-associated protein 1 (Keap1) and cytoplasmic Nrf2 form a complex. In reaction to oxidative stress, the protein Keap1 separates from Nrf2 and transfers to the nucleus, where it reacts with antioxidant response elements (AREs), initiating the transcription of genes such as “superoxide dismutase (SOD), heme oxygenase-1 (HO-1), and catalase (CAT)” (Raslan et al., 2021). Therefore, Nrf2 signaling stimulation reduces liver damage and fibrosis caused by toxins (Li et al., 2020).

Class III histone deacetylases, known as sirtuins, use one NAD+ molecule for each cycle of deacetylation (Imai et al., 2000). NAD-dependent protein deacetylase sirtuin 1 (SIRT1), a member of the sirtuin family, can directly deacetylate target proteins to drive various hepatic metabolic processes such as gluconeogenesis and lipid synthesis (Wan et al., 2019). Moreover, previous research has highlighted that triggering SIRT1 inhibits hepatic fibrosis by preventing liver inflammation (Zhao et al., 2018). SIRT1 reduces the inflammatory response by deacetylating the nuclear factor-κB (NF-κB) P65 subunit, which decreases NF-κB signal transmission (Lei et al., 2016; Nakazawa et al., 2017). Through the activation of AMP-activated protein kinase (AMPK) (a heterotrimeric serine/threonine kinase), SIRT1 inhibits oxidative stress by stimulating the translocation of Nrf2 (Ji et al., 2022), which, in turn, enhances its downstream antioxidant genes (Didamoony et al., 2022). Since activating AMPK dramatically enhances SIRT1 activity, it has been proven that SIRT1 and AMPK are intimately connected (Oliva et al., 2008). AMPK, a cellular energy status sensor, lowers ATP consumption, increases ATP synthesis, activates catabolic pathways, and inhibits many anabolic pathways to restore energy equilibrium (Towler and Hardie, 2007; Hardie, 2008). By increasing cellular NAD+ levels, AMPK increases SIRT1 activity. This deacetylates and modifies the activity of downstream SIRT1 targets (Cantó et al., 2009).

New oral antidiabetic medications, known as sodium–glucose co-transporter 2 inhibitors (SGLT2 I) can effectively treat type 2 diabetes mellitus (T2DM) by increasing renal glucose excretion through an insulin-independent mechanism (Frampton, 2018). Numerous investigations have shown that SGLT2 Is are a promising class of drugs for treating liver fibrosis (Grander et al., 2023). Because SGLT2 has hypoglycemic, antioxidant, and anti-inflammatory properties, which reduce oxidative stress and cytokine levels, causing inflammation, we thought repurposing it for liver injury may help to prevent liver fibrosis (Ozutsumi et al., 2020; Shaaban et al., 2022). By moving Nrf2 to the nucleus and initiating Nrf2/ARE signaling, SGLT2 Is lower oxidative stress and decrease fibrosis (Li et al., 2019). Remogliflozin (Remo) is an FDA-approved SGLT2 I (Markham, 2019). A novel SGLT2 I is remogliflozin, also known as remogliflozin etabonate. The efficacy of Remo in treating T2DM has been shown in numerous phase II clinical trials through its ability to reduce postprandial glucose excursions, improving HbA1c, improving insulin sensitivity, and enhancing pancreatic beta cell function, with clinically proven insulin-sensitizing properties (Nakano et al., 2015). Remogliflozin exhibits a significant degree of selectivity for SGLT2 over SGLT1 in contrast to previous SGLT Is (Wright et al., 2002). Nevertheless, the data regarding remogliflozin for the treatment of liver fibrosis is limited. Consequently, we aimed to investigate the potential protective effects of the novel oral hypoglycemic agent remogliflozin as a therapeutic intervention in the progression of liver fibrosis induced by TAA, along with an exploration of the underlying mechanisms.

## 2 Materials and methods

### 2.1 Animals

Twenty-four mature male Wistar rats, weighing 180–220 g and between 6 and 8 weeks old, were donated by the Animal House Colony at the National Research Centre (NRC, Egypt). The animals were kept under a 12-hour light/dark cycle at a temperature of 25°C.

### 2.2 Chemicals

TAA was bought from Sigma Aldrich, Burlington, MA, United States. Kissei Pharmaceutical Co., Ltd. (Matsumoto, Nagano, Japan) synthesized remogliflozin etabonate. The best available analytical-grade additional substances were used in this investigation.

### 2.3 Experimental protocol

Following 1 week of acclimation, six rats were allocated randomly to one of four groups: Rats in Group 1, the control group, were given saline intraperitoneally (IP) twice weekly for 6 weeks. To develop liver fibrosis, rats in Group 2, the TAA group, received IP injections of TAA (100 mg/kg b.wt), twice weekly for 6 weeks (Abd El-Rahman and Fayed, 2019). Groups 3 and 4 (the treated groups) were given two oral doses of Remo daily (25 or 50 mg/kg b.wt) for 4 weeks (Abdullah Ali et al., 2023) (the treatments were initiated 2 weeks after the TAA injections and continued concurrently with the TAA injections).

### 2.4 Preparation of blood and liver tissues

Twenty-four hours after the last injection, blood was obtained from the tail vein under anesthesia with intraperitoneal injections of ketamine (50 mg/kg) + xylazine (25 mg/kg) dissolved in saline. Euthanasia was performed using a CO_2_ euthanasia chamber, followed by decapitation. Liver samples were extracted directly and rinsed in ice-cold saline before being dried. Samples of serum were frozen at −20°C until used for biochemical analysis. A portion of each weighted rat’s liver was preserved at −80°C for research on molecules and biochemistry. A separate portion was preserved in 10% buffered neutral formalin for immunohistochemistry and histological analysis.

### 2.5 Assessment of the lipid profile and liver injury indicators

Triglycerides (TAGs) and total cholesterol (TC) were acquired from Cayman Chemical, Ann Arbor, MI, United States (Cat. Nos. 10010303 and 10007640, respectively), and albumin levels were measured using colorimetric techniques (GenWay Biotech, Inc. San Diego, CA, United States; Cat. No. GB0032). Colorimetric kits for aspartate aminotransferase (AST) and alanine aminotransferase (ALT) were acquired from Biomatik, Wilmington, DE, United States; their respective Cat. Nos were EKE62019 and EKU02211. Colorimetric quantifications were performed according to the manufacturer’s instructions for each kit.

### 2.6 Analysis of oxidative stress

SOD activity, glutathione (GSH) content, and malondialdehyde (MDA) levels (all of which are indicators of the oxidative stress burden) were measured in hepatic tissue homogenates using the colorimetric kits obtained from BioVision, Milpitas, CA, United States: SOD kit (Cat: K335–100), GSH kit (Cat: K464–100), and MDA kit (Cat: K739–100), according to the guidelines provided by the manufacturer.

### 2.7 ELISA tests for inflammation marker assay

Specific rat ELISA kits from SunLong Biotech Co., Ltd., Hangzhou City, Zhejiang, China (catalog numbers SL0722Ra, SL0411Ra, and SL0537Ra, respectively) were used to measure tumor necrosis factor-α (TNF-α), interleukin-6 (IL-6), and NF-κB in hepatic homogenates, which are key indicators of liver inflammation. At 450 nm, the optical density (OD) for the measured parameters was determined using spectrophotometry (Grellner et al., 2000).

### 2.8 ELISA tests for SIRT1, AMPK, p-AMPK, and Nrf2 levels

Levels of SIRT1 (Cat# SL1254Ra), AMPK (Cat# SL1695Ra), p-AMPK (Cat# SL0570Ra), and Nrf2 (Cat# SL0985Ra) in kidney tissues were assessed using the ELISA method, following the manufacturer’s protocol (SunLong Biotech Co., Ltd., Hangzhou City, Zhejiang, China).

### 2.9 Assay of hepatic Nrf2 and NF-κB gene expressions

#### 2.9.1 RNA extraction

The RNeasy QIAamp -Mini kit (Qiagen, Hilden, Germany) was used for the extraction of RNA from tissue samples after 30 mg of tissue was added to 600 µL of RLT buffer containing 10 μL ofβ-mercaptoethanol per 1 mL. In order to mechanically homogenize the tissue samples, the tubes were gently inserted into adapter sets secured to the Qiagen tissue homogenizer clamps. A 2-min adjustment to the highest-speed (30 Hz) shaking step induced disruption. Total RNA from the tissue was purified using the QIAamp RNeasy mini-kit. Then, 70% of the ethanol was added to the cleared lysate. It should be noted that DNase columns removed residual DNA.

#### 2.9.2 Primers

Primers obtained from Metabion, Germany, are listed in Table 1.

**TABLE 1:** List of the target genes, primer sequences, amplicon sizes, and cycling parameters for SYBR Green RT-PCR.

Gene	Forward or Backward primers	Sequence (5′–3′)
Nrf2 (Yamashita et al., 2014)	F	“CAC​ATC​CAG​ACA​GAC​ACC​AGT”
	R	“CTA​CAA​ATG​GGA​ATG​TCT​CTG​C”
NF-κB (Kermanian et al., 2013)	F	“GCA​AAC​CTG​GGA​ATA​CTT​CAT​GTG​ACT​AAG”
	R	“ATA​GGC​AAG​GTC​AGA​ATG​CAC​CAG​AAG​TCC”
Rat ß-actin (Banni et al., 2010)	F	“TCC​TCC​TGA​GCG​CAA​GTA​CTC​T”
	R	“GCT​CAG​TAA​CAG​TCC​GCC​TAG​AA”

#### 2.9.3 RT-PCR using SYBR Green

Primer testing was conducted using a 25-µL reaction mix that contained 12.5 µL of the 2x Quanti-Tect PCR SYBR Green Master Mix (Qiagen, Germany), 0.25 µL of Revert-Aid Reverse Transcriptase (200 U/µL) (Thermo Fisher), 0.5 µL of each primer of 20 pmol, 8.25 µL of water, and 3 µL of RNA template. A Stratagene MX3005P real-time PCR device was used to carry out the tests.

#### 2.9.4 Interpreting the SYBR Green real-time PCR findings

The Stratagene MX3005P program was used to determine each amplification curve and CT value using the delta–delta CT method described by Yuan et al. (2006).

### 2.10 Analysis of liver histology

Following the experiment, liver tissue samples were extracted from each group, cleansed, dehydrated, preserved in 10% neutral-buffered formalin, and embedded in paraffin. Following their sectioning at a thickness of 5 microns, the paraffin-embedded blocks were stained using hematoxylin and eosin (H&E) (Bancroft and Gamble, 2008) for examination.

#### 2.10.1 Lesion scoring in histopathology

The histological alterations in the liver were graded using the following percentage system: no changes (0), mild changes (1), perisinusoidal or periportal and mild perisinusoidal changes (2), bridging fibrosis (3), and cirrhosis (4). Changes of less than 30% were deemed mild, those between 30% and 50% were deemed moderate, and those beyond 50% were deemed severe (Kleiner et al., 2005; Shamseldean et al., 2022).

### 2.11 Immunohistochemical evaluation

Liver tissue sections were deparaffinized in xylene and then rehydrated in graded alcohols (El-Maksoud et al., 2020). The sections were pretreated for 20 min with a citrate buffer solution at pH 6 to achieve antigen retrieval. A humidified chamber was used to incubate the sections for 2 hours with a mouse monoclonal antibody against rabbit polyclonal NF-kB p65 antibody (ab16502; 1:1000 dilution, Abcam, Cambridge, UK) and SIRT1 (ab110304; 1:100 dilution, Abcam, Cambridge, UK). The sections were then incubated with goat anti-rabbit IgG H&L (HRP) (ab205718; Abcam, Cambridge, UK) and chromogenized with 3,3′-diaminobenzidine tetrahydrochloride (DAB, Sigma). The slides were mounted using DP and counterstained with hematoxylin. Negative control slides were created using PB by replacing the primary antibodies.

#### 2.11.1 Assessment of SIRT1 and NF-kB p65 immunostaining

Five tissue slices were evaluated for SIRT1 and NF-kB p65 quantitative immunoreactivity in each group. Each segment’s immunoreactivity was examined using a (x 400) high-power microscope in 10 microscopical areas. The ImageJ 1.52 p color deconvolution program (Wayne Rasband, National Institutes of Health, United States) was used to calculate the proportion of positively labeled cells (%).

### 2.12 Statistics of the experiment

The statistical analysis was performed in accordance with Elbaset et al. (2023b). The Shapiro test ensured that the values were normally distributed, and the Brown–Forsythe test was used to check for heteroscedasticity. The results are shown as the mean ± standard error. A one-way analysis of variance (ANOVA) and the Tukey–Kramer *post hoc* test were used to process the data. Meanwhile, nonparametric data were presented as he median ± interquartile range and analyzed using the Kruskal–Wallis test, followed by Dunn’s test. Figures were generated, and statistical analyses were carried out using GraphPad Prism (version 10, California, United States). All statistical tests were conducted with a significance threshold of p < 0.05.

## 3 Results

### 3.1 Impact of remogliflozin on liver function markers in TAA-intoxicated rats

**FIGURE 1:**
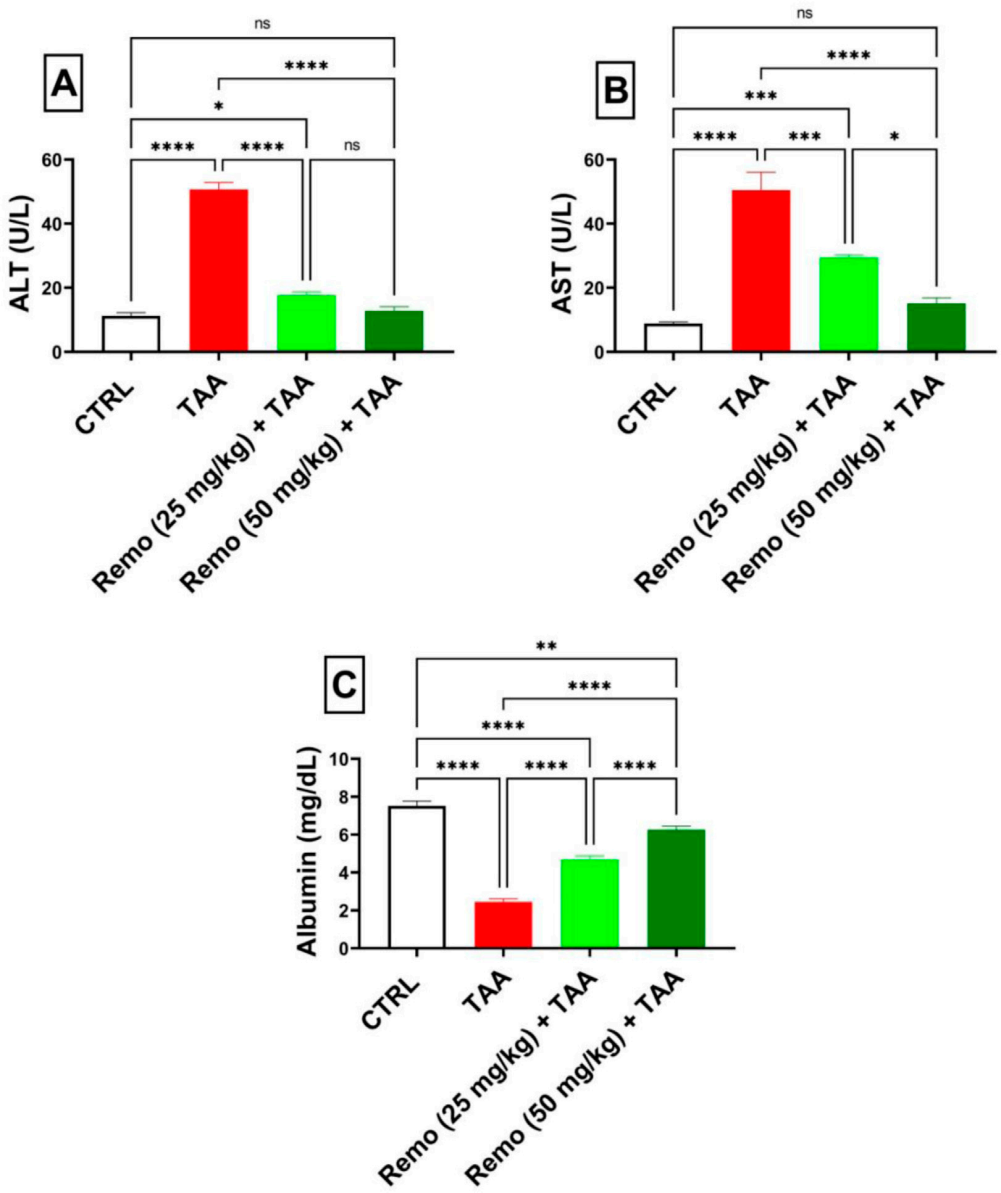
Assessment of the remogliflozin impact on liver function: AST, ALT, and albumin in TAA-intoxicated rats. **(A)** “Serum ALT” (U/L). **(B)** “Serum AST” (U/L). **(C)** “Serum albumin” (mg/dL). Data are displayed as the mean ± SEM of six rats, and an asterisk represents the statistical significance between pairwise comparisons. TAA, thioacetamide; Remo, remogliflozin.

TAA administration significantly impaired liver function, as evidenced by a 350.9% increase in ALT and a 472.4% increase in AST, while albumin decreased by 67.4%, in contrast to the control group. Administration of Remo (25 mg/kg) significantly improved these parameters by reducing ALT and AST by 65% and 41.6%, respectively, while increasing albumin by 91.6% compared to the TAA group. The higher dose (50 mg/kg) showed superior effects, reducing ALT and AST by 74.7% and 70%, respectively, while increasing albumin by 155.6% compared to the TAA group, bringing these parameters closer to normal values (Figures 1A–C).

### 3.2 Impact of remogliflozin on oxidative stress in TAA-intoxicated rats

**FIGURE 2:**
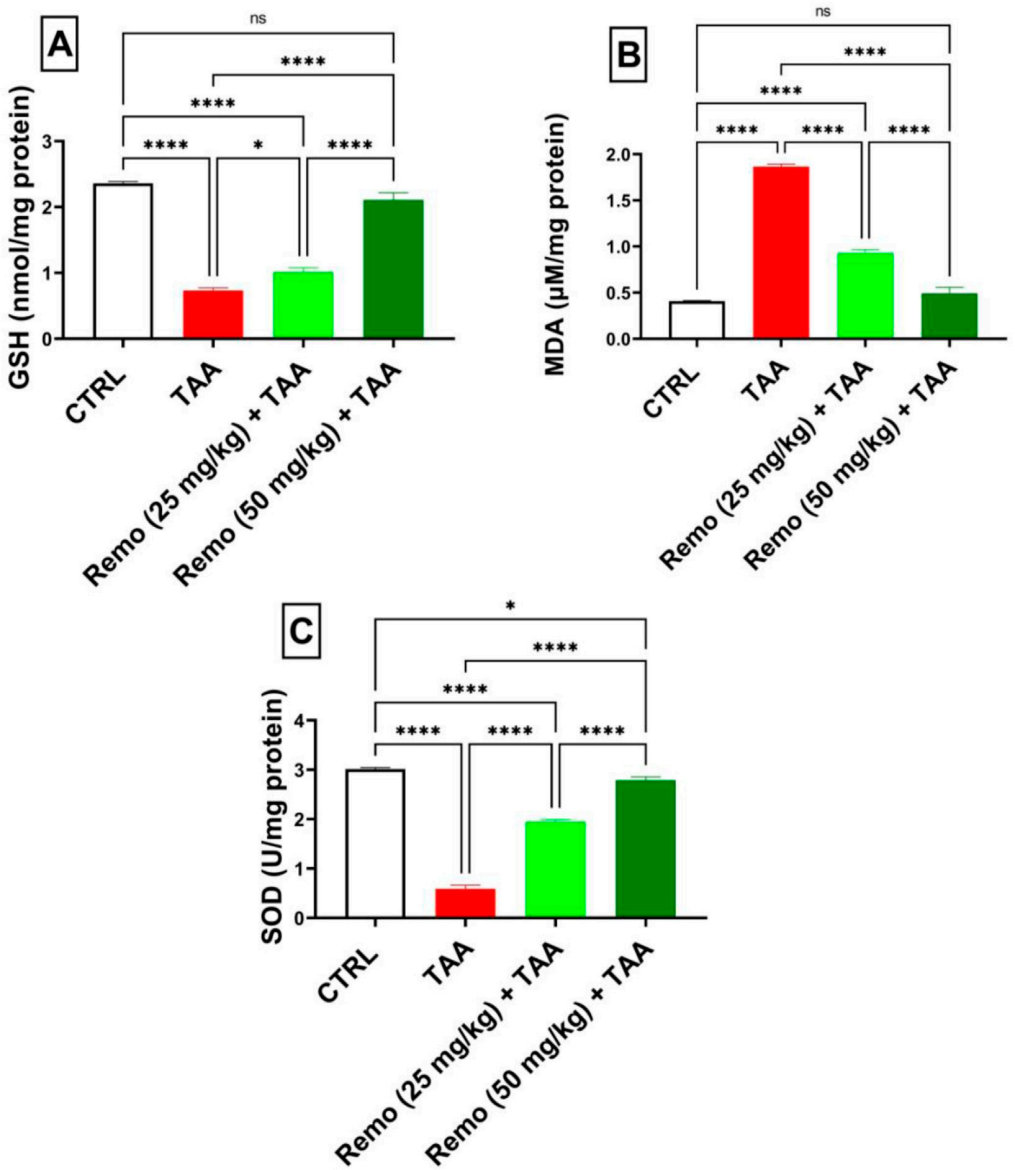
Remogliflozin’s impact on oxidative stress markers: GSH, MDA, and SOD in TAA-intoxicated rats. **(A)** GSH (nmol/mg protein), **(B)** MDA (µM/mg protein), **(C)** SOD (U/mg protein) activity. The data are displayed as the mean ± SEM of six rats, and an asterisk represents the statistical significance between pairwise comparisons at p < 0.05. TAA, thioacetamide; Remo, remogliflozin.

TAA induced severe oxidative stress by decreasing GSH and SOD by 69.1% and 80.3%, respectively, while increasing MDA by 359.1%, compared to the control group. Remo treatment dose-dependently improved these markers. In comparison with the TAA group, the 25-mg/kg dose reduced MDA by 50.2% and increased GSH and SOD by 38.8% and 229.2%, respectively. In comparison to the TAA group, the benefits of the 50-mg/kg dose were more noticeable, increasing GSH and SOD by 189% and 370.9%, respectively, and reducing MDA by 73.7%, nearly normalizing these parameters (Figures 2A–C).

### 3.3 Impact of remogliflozin on lipid profiles in TAA-intoxicated rats

**FIGURE 3:**
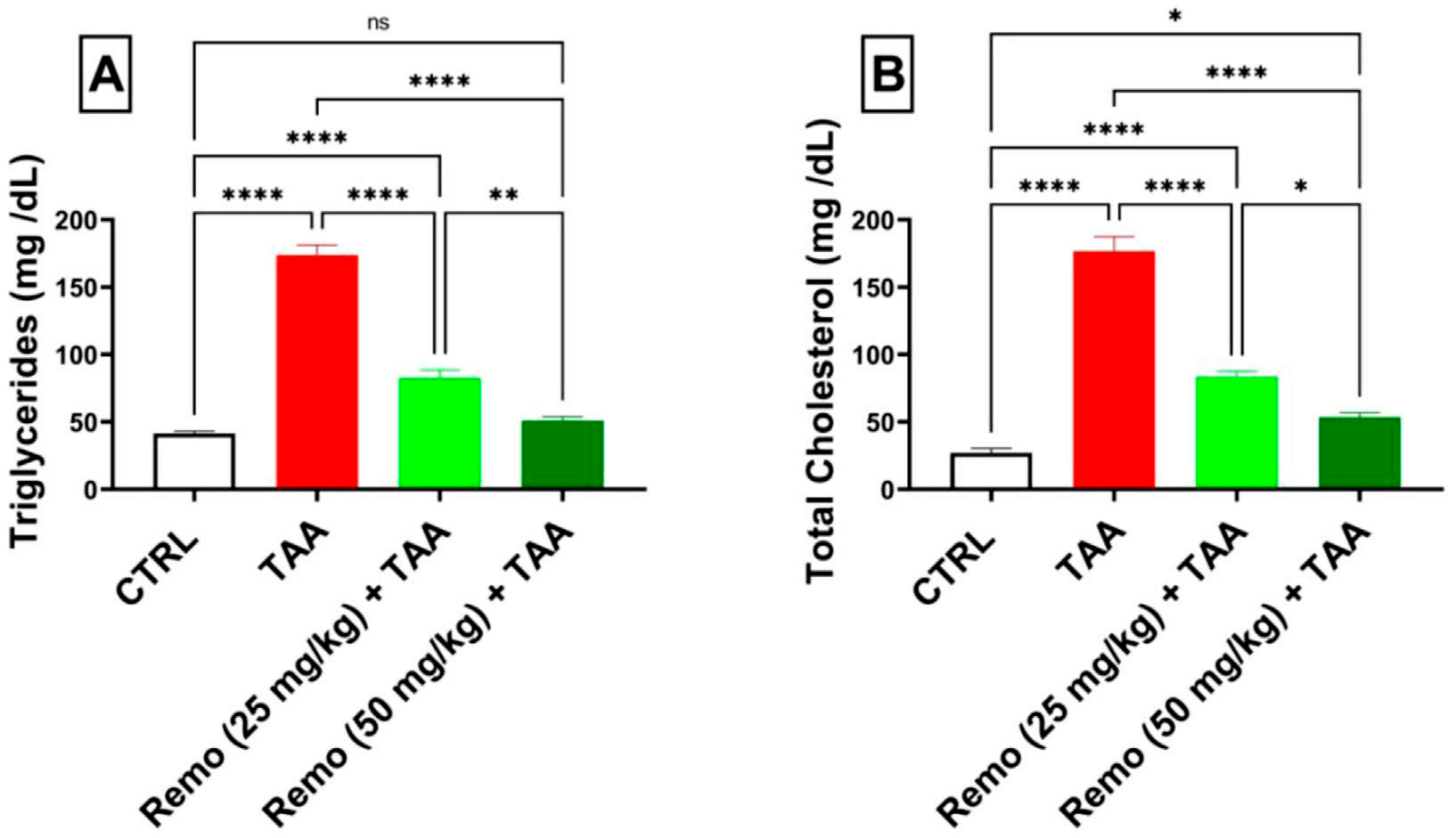
Assessment of remogliflozin’s effect on the lipid profile: serum triglycerides and total cholesterol in TAA-intoxicated rats. **(A)** Serum triglycerides (mg/dL). **(B)** Serum total cholesterol (mg/dL). The data are displayed as the mean ± SEM of six rats, and an asterisk represents the significance between pairwise comparisons at p < 0.05. TAA, thioacetamide; Remo, remogliflozin.

TAA administration significantly disturbed the lipid profile, increasing triglyceride and total cholesterol levels by 317.9% and 551.8%, respectively, compared to the control group. Remo treatment showed a dose-dependent improvement, with the 25-mg/kg dose reducing both markers by approximately 52.5%. In comparison, the 50-mg/kg dose displayed superior effects, reducing triglyceride and cholesterol levels by 70.7% and 69.8%, respectively, compared to the TAA group, bringing them closer to normal values (Figures 3A,B).

### 3.4 Impact of remogliflozin on inflammatory markers in TAA-intoxicated rats

**FIGURE 4:**
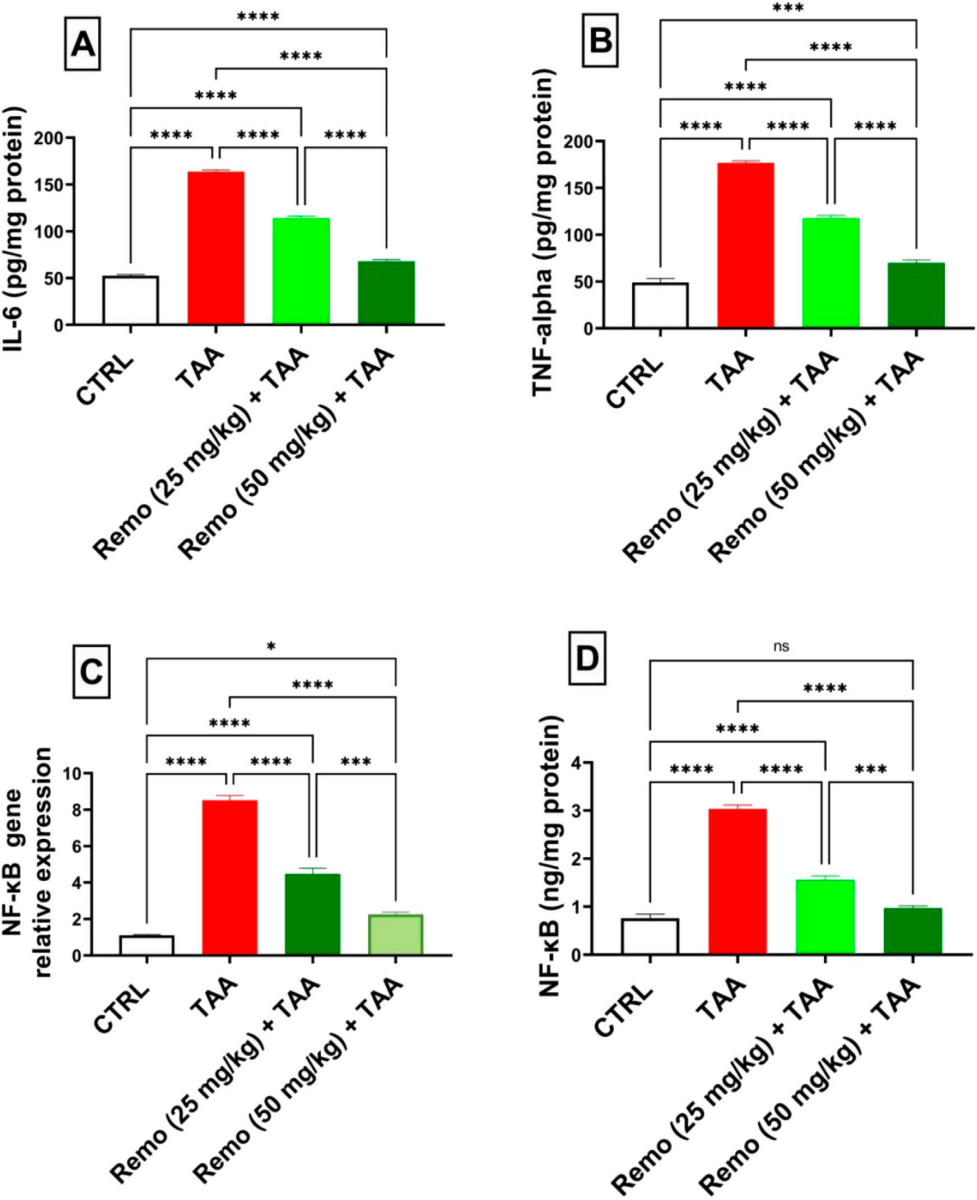
Remogliflozin’s effect on inflammatory mediators: IL-6, TNF-α, and NF-κB gene expression and levels in TAA-intoxicated rats. **(A)** IL-6 (pg/mg protein). **(B)** TNF-α (pg/mg protein). **(C)** NF-κB gene expression. **(D)** NF-κB (ng/mg protein). The data are displayed as the mean ± SEM of six rats, and an asterisk displayed on the bars represents the significance between pairwise comparisons at p < 0.05. TAA, thioacetamide; Remo, remogliflozin.

TAA induced significant inflammation, increasing NF-κB protein, IL-6, TNF-α, and NF-κB gene expressions by 298.5%, 211.9%, 259.7%, and 669.9%, respectively, compared to the control group. Remo treatment dose-dependently reduced these inflammatory markers. The 25-mg/kg dose reduced these parameters by 48.8%, 30.3%, 33.4%, and 47.5%, respectively, while the 50-mg/kg dose showed more pronounced effects, reducing them by 67.9%, 58.6%, 60.4%, and 73.6%, respectively, in comparison with the TAA group (Figures 4A–D).

### 3.5 Impact of remogliflozin on energy sensors in TAA-intoxicated rats

**FIGURE 5:**
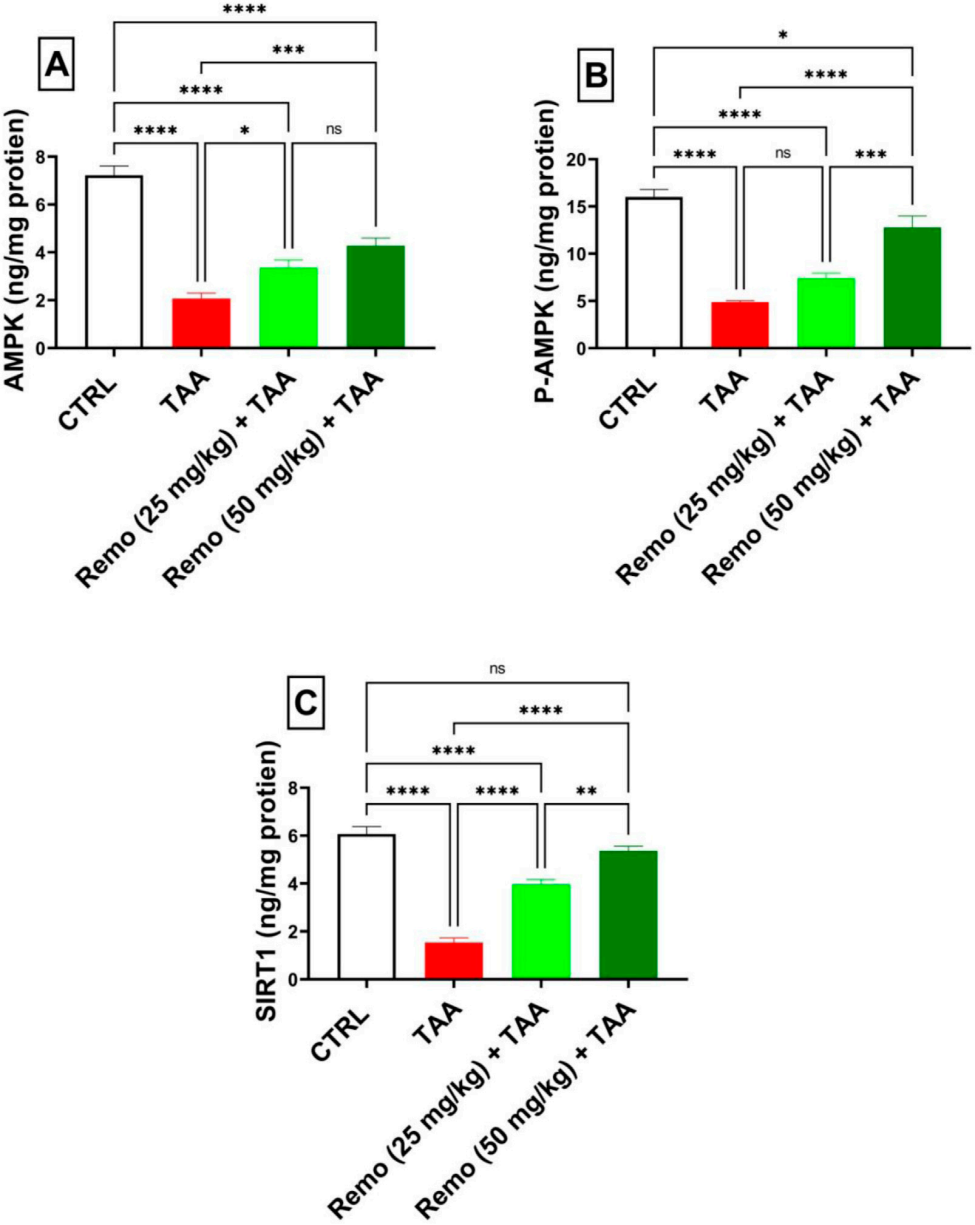
Remogliflozin’s effect on energy sensors: AMPK, P-AMPK-α, and SIRT1 in TAA-intoxicated rats. **(A)** “AMPK (ng/mg protein).” **(B)** “P-AMPK (ng/mg protein).” **(C)** “SIRT1 (ng/mg protein).” The data are displayed as the mean ± SEM of six rats, and an asterisk displayed on the bars represents the significance between pairwise comparisons at p < 0.05. TAA, thioacetamide; Remo, remogliflozin.

TAA significantly decreased energy sensors, reducing AMPK, P-AMPK, and SIRT1 by 71.4%, 69.5%, and 74.7%, respectively, compared to the control group. Remo treatment showed dose-dependent improvement. The 25-mg/kg dose increased these parameters by 62.2%, 51.7%, and 158.5%, respectively. In comparison, the 50-mg/kg dose displayed superior effects, increasing them by 107.1%, 162%, and 249.3%, respectively, compared with the TAA group, bringing them closer to normal values (Figures 5A–C).

### 3.6 Impact of remogliflozin on the Nrf2 pathway in TAA-intoxicated rats

**FIGURE 6:**
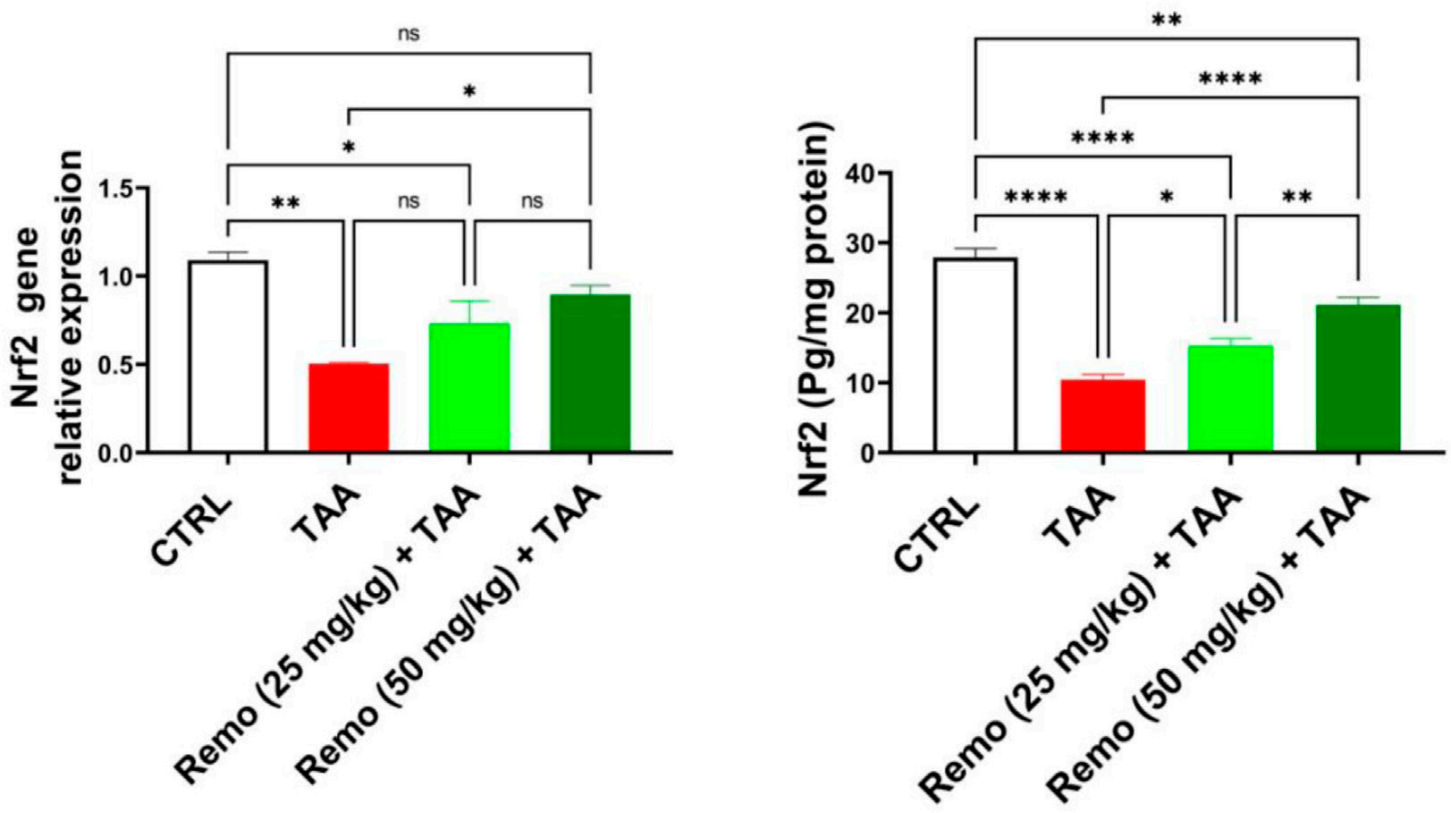
Remogliflozin’s effect on Nrf2 gene expression and level in TAA-intoxicated rats. The data are displayed as the mean ± SEM of six rats, and an asterisk displayed on the bars represents the significance between pairwise comparisons at p < 0.05. TAA, thioacetamide; Remo, remogliflozin.

Compared to the control group, TAA significantly suppressed the Nrf2 pathway by reducing Nrf2 protein and gene expressions by 62.7% and 53.8%, respectively. Remo treatment dose-dependently enhanced this pathway. The 25-mg/kg dose increased Nrf2 protein and gene expressions by 46.7% and 45.4%, respectively. In comparison, the 50-mg/kg dose displayed more pronounced effects, increasing them by 103.1% and 77.8%, respectively, in contrast to the TAA group, approaching normal values (Figure 6). It is mentioned in all graphs whether the reduction or increase is significant or not.

### 3.7 Results of histopathology

**FIGURE 7:**
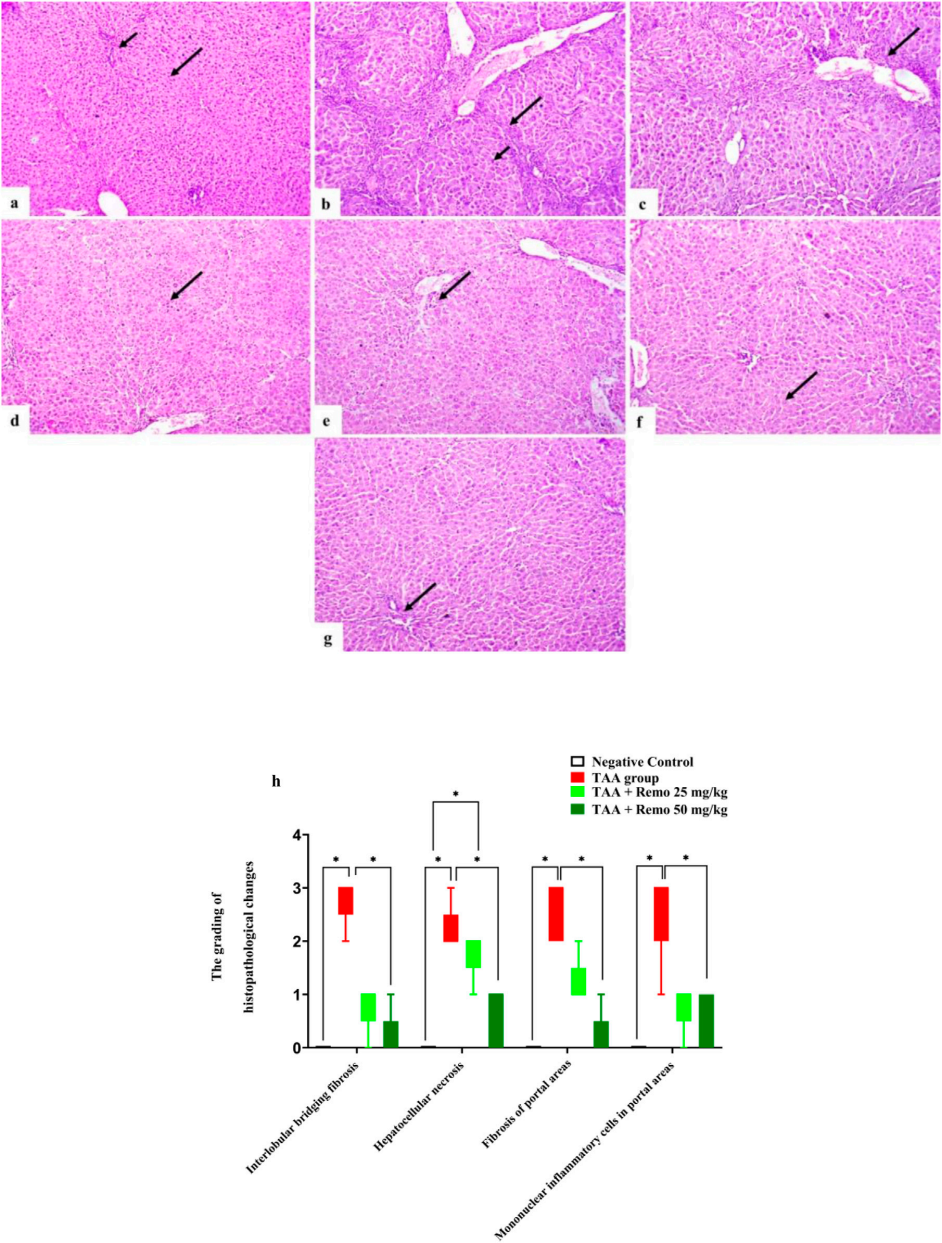
Rat liver photomicrograph (H&E X100). **(a)** The negative group exhibits a normal portal area (short arrow) and a normal hepatocyte histological structure (long arrow). **(b)** The TAA group has hepatocellular necrosis (short arrow) and interlobular bridging fibrosis (long arrow). **(c)** The TAA group displays portal region infiltration of mononuclear inflammatory cells and fibrosis (arrow). **(d)** The TAA + Remo 25 mg/kg group shows some hepatocyte necrosis (arrow). **(e)** Mild portal fibrosis and mononuclear inflammatory cell infiltration are visible in the TAA + Remo 25 mg/kg group (arrow). **(f)** Hepatocytes in the TAA + Remo 50 mg/kg group appear almost normal (arrow). **(g)** Normal portal regions are displayed in the TAA + Remo 50 mg/kg group (arrow). **(h)** The grading of histopathological changes. Each whisker represents the median and interquartile range. An asterisk on the bars represents the significance between pairwise comparisons at p < 0.05. TAA, thioacetamide; Remo, remogliflozin.

The control group’s hepatocyte and portal area histological structures were normal (Figure 7a). The TAA group exhibited interlobular bridging fibrosis with hepatocellular necrosis (Figure 7b) along with fibrosis and mononuclear inflammatory cell infiltration of the portal areas (Figure 7c). The TAA + Remo 25 mg/kg group showed necrosis of some hepatocytes (Figure 7d), mild portal fibrosis, and mononuclear inflammatory cell infiltration (Figure 7e). The TAA + Remo 50 mg/kg group showed nearly normal hepatocytes (Figure 7f) and normal portal areas (Figure 7g).

A score was determined for the histopathological liver lesions. As indicated in Figure 7h, each observed liver lesion was graded based on severity.

### 3.8 Results of liver NF-kB p65 and SIRT1 immunohistochemistry

**FIGURE 8:**
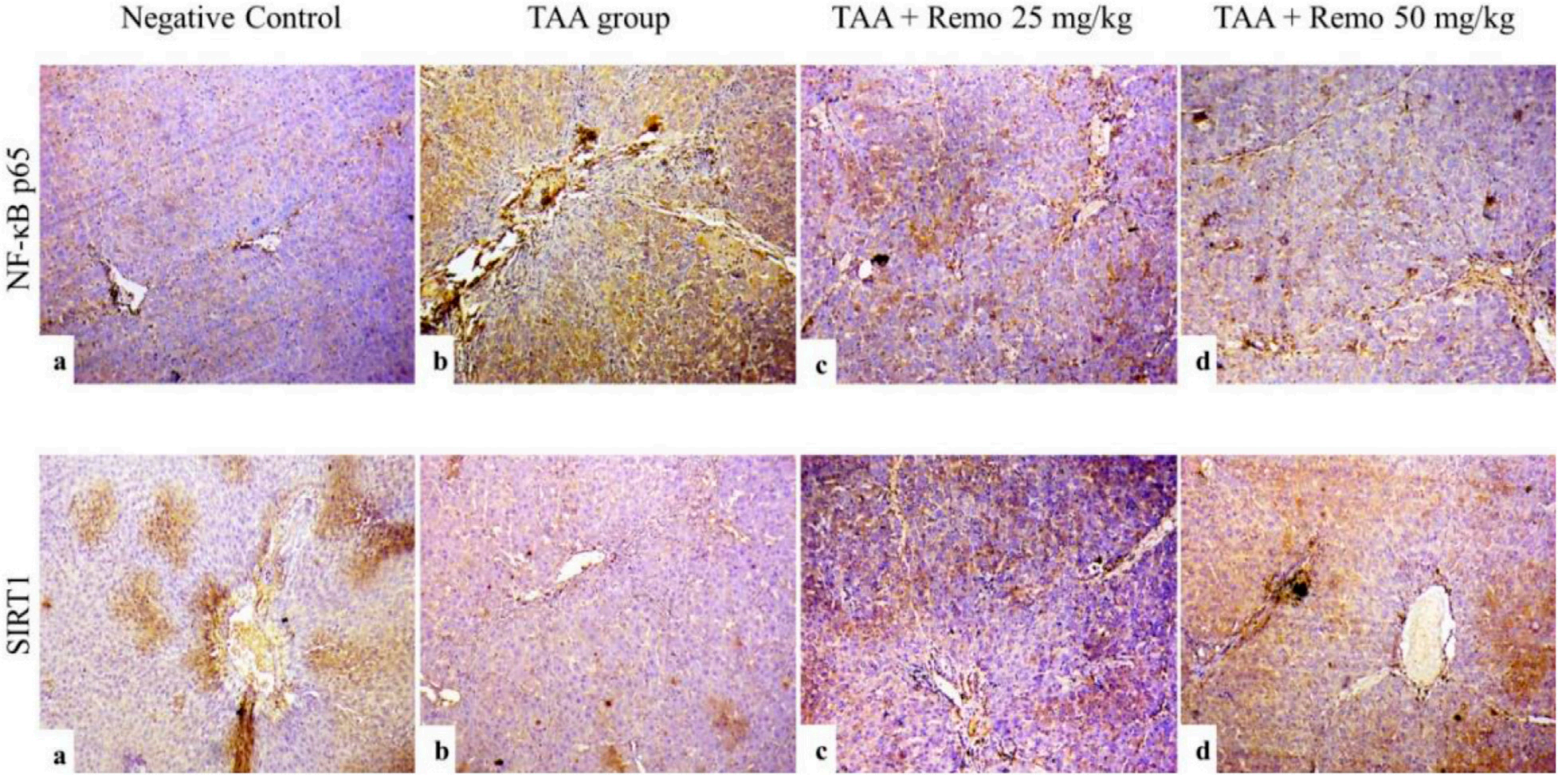
Immunostaining of NF-kB p65 and SIRT1 in the rat liver (X100), **(a)** SIRT1 expression is high, and NF-kB p65 immune expression is minimal in the control group. **(b)** The TAA group exhibits low SIRT1 expression and high NF-kB p65 immunoreactivity. **(c)** The TAA + Remo 25-mg/kg group shows a higher SIRT1 immune-staining response and modest NF-kB p65 immunoreactivity. **(d)** The TAA + Remo 50-mg/kg group shows robust immune-staining of SIRT1 and weak immune-expression of NF-kB p65. (data are expressed as mean ± SE; an asterisk on the bars represents the significance between pairwise comparisons at p < 0.05). TAA, thioacetamide; Remo, remogliflozin.

**FIGURE 9:**
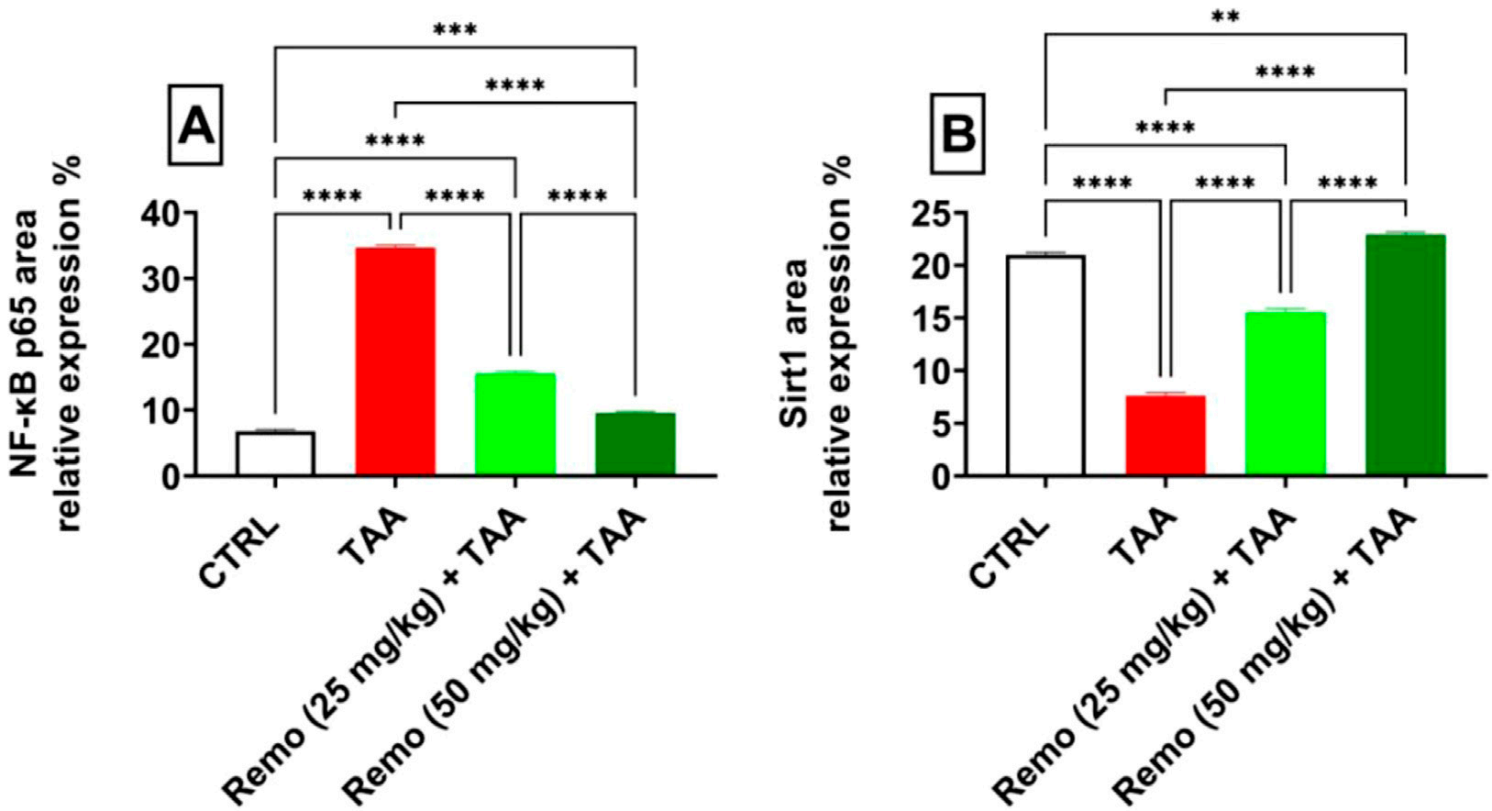
Immunostaining expression is % of **(A)** NF-kB p65 and **(B)** SIRT1 in the liver (data are expressed as mean ± SE; an asterisk displayed on the bars represents the significance between pairwise comparisons at p < 0.05). TAA, thioacetamide; Remo, remogliflozin.

SIRT1 was strongly expressed and NF-kB p65 was weakly expressed in the control group (Figure 8a). SIRT1 expression was weak. NF-kB p65 immunoreactivity was significant in the TAA group (Figure 8b). The TAA + Remo 25 mg/kg group demonstrated a substantial increase in SIRT1 immune-staining response and modest positivity of NF-kB p65 (Figure 8c). The TAA + Remo 50 mg/kg group demonstrated robust immune-staining of SIRT1 and weak immune-expression of NF-kB p65 (Figure 8d). NF-kB p65 and SIRT1 % area immunostaining expressions in different groups’ liver tissues are shown in Figure 9.

## 4 Discussion

An organosulfur compound, thioacetamide (C2H5NS), is a commonly used animal model for acute and chronic liver injury that closely mimics human liver fibrosis (Abdel-Rahman and Fayed, 2021). TAA-sulfur dioxide (TAA-S-dioxide), a highly dangerous reactive metabolite that adheres to liver macromolecules and parenchyma to cause necrosis and activate HSCs, is created when CYP450 converts TAA (Megahed et al., 2023). Cellular membranes become weaker and more permeable due to TAA-S-dioxide’s breakdown, which allows the liver enzymes AST and ALT to leak into the blood circulation (Abdel-Rahman et al., 2023; Elbaset et al., 2023a). A marked upsurge in liver enzymes and a sharp decrease in albumin levels indicated the detrimental effects of TAA on liver cells, according to the results of our investigation. These results agreed with previous research findings (Chen et al., 2021; Aslam et al., 2022; Elbaset et al., 2023b). Restoration of serum albumin levels and decreased enzymatic activity indicate that Remo treatment successfully maintains hepatocyte integrity in a dose-dependent manner. Our findings demonstrate that Remo shields the liver against TAA toxicity. In line with our findings, a study by Nakano et al. (2015) demonstrated that Remo enhanced NAFLD-related markers in male mice that were made obese by diet.

Under typical physiological circumstances, oxidative stress generation and clearance are controlled by “endogenous antioxidant defense systems, which comprise antioxidant enzymes (SOD) and non-enzymatic (GSH)” (Liao et al., 2022). Antioxidants in hepatocytes are depleted or rendered inactive due to an imbalance in the pro-oxidant-to-antioxidant ratio, which is the ultimate cause of many hepatic diseases (Koyama et al., 2014). We found that TAA significantly increased oxidative stress, as evidenced by a considerable elevation in liver MDA and a marked decrease in SOD activity and GSH content. These outcomes align with those of previous studies (El-Mihi et al., 2017; Özdemir-Kumral et al., 2019). We evaluated Remo’s antioxidant capacity against oxidative stress by enhancing SOD and GSH levels and inhibiting lipid peroxidation in the groups that received Remo treatment. Remogliflozin is an O-linked glycoside; this is a characteristic feature of its chemical structure, as the other SGLT2 inhibitors are C-linked glycosides. This suggests that Remo may possess intrinsic antioxidant properties. In line with our results, a study by Abdullah Ali et al. (2023) demonstrated that Remo prevented oxidative damage in rats with streptozotocin-induced diabetes mellitus. Remo’s insulin-sensitizing and antioxidant qualities may make it a useful treatment for nonalcoholic steatohepatitis (NASH) and nonalcoholic fatty liver disease (NAFLD), according to a study conducted by Nakano et al. (2015).

The Nrf2 pathway is a crucial system that regulates inflammation and oxidative damage brought on by TAA. Nfr2 and antioxidant genes support a protective system that suppresses inflammation and oxidative damage (Chen et al., 2019). Certain genes involved in antioxidant defense, such as SOD, CAT, HO-1, and GSH, are activated, and their expression is controlled by the Nrf2–antioxidant response element (ARE) pathway (Alsharif et al., 2022). According to this study, the groups receiving Remo treatment had higher Nrf2 expression and levels, while these were reduced in the TAA group. Another study by Abdel-Rahman et al. (2022) showed that TAA inhibited the NRF2/ARE signaling pathway in a rat model of experimentally induced liver fibrosis, which is consistent with our findings. Meanwhile, it was documented that SGLT2 inhibition enhances Nrf2 activation (Kim et al., 2021). Consequently, Remo’s defense against TAA-induced liver fibrosis may consist of an elevation in nuclear Nrf2 expression, which also increases endogenous antioxidant defense. These results demonstrate that Remo’s antifibrotic actions are significantly mediated by the Nrf2 pathway.

The nuclear respiratory factors (Nrf1 and Nrf2) that initiate the transcription of genes linked to mitochondrial biogenesis are co-activated by SIRT1 through the activation and deacetylation of peroxisome proliferator-activated receptor gamma coactivator 1 alpha (PGC-1α) (Scarpulla, 2011), a master regulator of mitochondrial biogenesis. AMPK, another crucial metabolic sensor, also activates PGC-1α (Jäger et al., 2007). SIRT1 and AMPK share several roles, including reacting to nutritional status and stress, initiating mitochondrial biogenesis, regulating the activity of important transcriptional regulators such as PGC-1α, and managing glucose homeostasis (Fulco and Sartorelli, 2008). Additionally, it has been shown that AMPK indirectly activates SIRT1 by increasing the intracellular concentrations of its co-substrate, NAD+ (Lan et al., 2008). AMPK activation can enhance the expression of genes associated with antioxidant defense mechanisms (Essick and Sam, 2010). Interestingly, nuclear accumulation of the antioxidant transcription factor Nrf2 is connected to AMPK activation (Joo et al., 2016). SIRT1 aids in reducing proinflammatory cytokines, increasing antioxidant genes, and altering Nrf2/NF-κB crosstalk when oxidative damage occurs. Furthermore, SIRT1/AMPK signaling controls inflammatory transcription factors, such as NF-κB, which is the main modulator of several proinflammatory cytokines (Nagappan et al., 2019; Mansoure et al., 2024). AMPK suppresses NF-κB expression, which lowers the inflammatory response by upregulating SIRT1 expression (Chen et al., 2016). According to recent data, SIRT1 activation protects against liver fibrosis and inflammation (Zhao et al., 2018). AMPK controls the inflammatory response, preserves cellular energy balance, and plays a key role in the transformation of macrophages from a proinflammatory to an anti-inflammatory phenotype during inflammation (Velagapudi et al., 2017). According to this study’s data, TAA intoxication reduces the levels of hepatic SIRT1 and AMPK, along with active AMPK (p-AMPK), compared to the control group. In agreement with our results, a study by Shin et al. (2021) showed that *Gardeniae Fructus* reduces TAA-induced liver fibrosis in mice by activating the Nrf2 and AMPK/SIRT1 pathways. Remo therapy, however, increased the levels of the aforementioned parameters. To our knowledge, no research has investigated how Remo treatment affects SIRT1, AMPK, and p-AMPK levels in TAA-induced hepatic fibrosis. According to Zhou et al. (2018), SGLT2 inhibitor-mediated SIRT1/AMPK signal transduction activation can lower oxidative stress, restore mitochondrial structure and function, and reduce inflammation, which confirms our findings. These findings support the hypothesis that Remo’s hepatoprotective activity against TAA-induced liver fibrosis is partially due to stimulation of SIRT1/AMPK signaling.

Previous research has shown that chronic liver injury and inflammation manifest before liver fibrosis and can reinforce one another (Alshanwani et al., 2021). Excessive inflammatory reactions are strongly linked to the inhibition of antioxidant defense systems and the increased oxidative stress in hepatocytes (Fawzy et al., 2023). A major factor in developing liver fibrosis is NF-κB signaling, which is crucial for controlling inflammation. By blocking the NF-κB signaling pathway, liver tissue can exhibit reduced expression of proinflammatory cytokines (IL-6 and TNF-α), in addition to decreased collagen deposition, fibrosis marker α-SMA expression, and liver fibrosis (Luedde and Schwabe, 2011; Aoyama et al., 2021). TNF-α, IL-6, and interleukin-1 beta (IL-1β) are proinflammatory cytokines that work in a vicious cycle with NF-κB signaling pathways to promote one another and intensify the inflammatory response, causing liver fibrosis (Hoesel and Schmid, 2013; Sun, 2017). The present study’s findings demonstrate that NF-κB/TNF-α/IL-6 levels were elevated, along with increased NF-κB p65 expression, following TAA injections. In agreement with our results, Abdelmageed and Abdelrahman (2023) demonstrated that TAA increased NF-κB, TNF-α, and IL-6 levels in a model of TAA-induced liver damage. However, Remo has been shown to lower NF-κB, NF-κB p65, and proinflammatory factors (IL-6 and TNF-α). In many inflammatory settings, Remo’s ability to suppress NF-κB and its downstream cytokines has been displayed (Nakano et al., 2015; Abdullah Ali et al., 2023). The results indicate that Remo may have an anti-inflammatory effect against TAA-induced liver fibrosis by modulating the NF-κB signaling pathway. In this work, histological analysis has confirmed that Remo has the potential to hinder the deleterious effects of TAA.

The current investigation used a preventive approach, in which remogliflozin was provided after the commencement of TAA exposure but before the development of advanced fibrosis. Consequently, our data primarily endorse the potential of remogliflozin as a prophylactic medication against the advancement of liver fibrosis rather than as a therapeutic intervention for existing illness. This difference is vital since the majority of clinical instances pertain to individuals with pre-existing fibrosis, necessitating curative rather than preventive measures. Moreover, the translational applicability of our results is constrained by the model’s preventive characteristics. Future research should use therapeutic models in which remogliflozin therapy starts upon the confirmation of developed fibrosis to more accurately evaluate its clinical significance and therapeutic effectiveness.

The mechanism of action indicates that SGLT2 is predominantly expressed in the kidney, with less expression in hepatic tissue (Bonner et al., 2015; Vallon, 2015). Consequently, the hepatic effects of remogliflozin may not stem from the direct inhibition of hepatic SGLT2. Remogliflozin’s hepatoprotective benefits may be delivered indirectly via systemic metabolic enhancements, including improved glycemic management, less insulin resistance, and lower systemic inflammation, thereby promoting liver function. This idea has been corroborated by previous research that demonstrated the systemic metabolic advantages of SGLT2 inhibitors in diabetic animals.

In conclusion, our study demonstrates the preventive effectiveness of remogliflozin in early-stage liver fibrosis. However, more research is required to assess its therapeutic potential in advanced illness and clarify the specific mechanisms of its hepatoprotective benefits. We recommend that further studies use a therapeutic paradigm, initiating remogliflozin treatment post-fibrosis development, and include direct evaluations of hepatic stellate cell activation alongside specialized histological stains for enhanced fibrosis quantification accuracy.

## 5 Conclusion

The study’s findings offer a new viewpoint on Remo’s hepatoprotective properties in cases of liver fibrosis. Remo has been successfully associated with regulating the SIRT1/AMPK/Nrf2/NF-кB signaling pathways, which improves the hepatic tissue’s antioxidant capacity and reduces inflammation. Remo has few side effects and is now safe and tolerable. According to our findings, Remo may be a good option as a protective treatment for early liver fibrosis. However, the current study’s use of Remo as a therapeutic agent to prevent liver fibrosis may be considered a limitation. More research is necessary to further examine this hypothesis.

### 5.1 Limitations

This study has several limitations. First, the experimental design did not include a remogliflozin-only group, which limited our ability to distinguish the direct effects of remogliflozin from its interaction with TAA-induced pathology or potential vehicle effects. Second, the preventive paradigm of commencing remogliflozin medication before the onset of severe fibrosis does not accurately represent the clinical reality, since patients generally present with developed fibrosis. However, it elucidates whether preventive action, if implemented upon early diagnosis, is effective. Third, the research failed to directly evaluate hepatic stellate cell activity or use special histology stains, such as Masson’s trichrome or Sirius Red, for enhanced fibrosis quantification. Fourth, the molecular connection between SGLT2 inhibition and the activation of the hepatic AMPK/SIRT1/Nrf2 pathway is currently unclear, and indirect systemic effects cannot be ruled out. Finally, the study included only male rats, which does not reflect the realm of liver fibrosis affecting both male and female rats.

### 5.2 Future directions

Future research should address these shortcomings by exclusively using remogliflozin and using proven fibrosis scoring methods with specialized stains for fibrosis evaluation. Mechanistic investigations using pathway inhibitors or transgenic models are essential to elucidate the causal link between SGLT2 inhibition and hepatic signaling pathways. Future studies should also use therapeutic models in which remogliflozin therapy starts post-establishment of liver fibrosis to more accurately evaluate its clinical significance as a prospective treatment. Furthermore, pharmacokinetic investigations are necessary to ascertain remogliflozin levels in hepatic tissue and to examine whether direct hepatic mechanisms or systemic metabolic enhancements facilitate its hepatoprotective effects. Ultimately, the practical use of remogliflozin necessitates collecting real-world information about analogous clinical situations of early fibrosis for future clinical use.

## Data Availability

The raw data supporting the conclusions of this article will be made available by the authors without undue reservation.
